# 109 Optimising Community-Based Physical Activity for Chronic Condition Management in Ireland: A Comprehensive Systems Analysis guided by RE-AIM

**DOI:** 10.1093/eurpub/ckae114.176

**Published:** 2024-09-26

**Authors:** Rachael O’Connor, Aisling McGrath, Clare Lodge, Niamh Murphy

**Affiliations:** South East Technological University, Ireland; South East Technological University, Ireland; South East Technological University, Ireland; South East Technological University, Ireland

## Abstract

**Purpose:**

The prevalence of chronic conditions (CC) is rising rapidly and coupled with an aging demographic, poses a significant threat to the well-being of populations and overburdened healthcare systems. The negative impact of CC on physical, mental, and social well-being requires innovative approaches to combat decline in quality of life (QOL). The concept of “exercise as medicine” demonstrates the value of physical activity (PA) in treating CCs. Community-based PA initiatives are accessible solutions to improve QOL and prevent disease progression in CC populations, yet contextual factors impacting implementation are not widely understood. Therefore, as part of a broader study, this research will provide a comprehensive qualitative systems analysis of community-based PA interventions for populations with CC in Ireland.

**Methods:**

This study will employ a qualitative data approach guided by systems approaches and implementation science. Following a desk-based review, semi-structured interviews guided by the RE-AIM framework will be conducted with multiple stakeholders to inform a comprehensive understanding of the landscape (intricacies and contextual factors) of community-based PA for populations with CC. Purposive and snowball sampling will recruit diverse stakeholders (providers, referrers, policymakers, funders) across sites. A hybrid coding approach of inductive and deductive coding guided by implementation science frameworks will be used for analysis of qualitative data.

**Results:**

This study aims to understand the key components and interconnections within the current system of community-based PA for CC in Ireland. This study will provide insights into barriers and facilitators influencing effectiveness and sustainability of community-based PA initiatives and aid in identifying implementation strategies to enhance the pathway of care into such initiatives for individuals with CC in Ireland.

**Conclusions:**

To ensure effective implementation of community-based PA programmes, a clear understanding of the overall landscape, relationships, interdependencies is required. Implications of this study will inform the broader project through identifying barriers influencing effective implementation of community-based programmes. This process will contribute to identifying a suitable delivery model for these programmes, leading to scale-up of facilities, enhancing QOL of individuals.

**Funding source:**

Funded by SETU Co-fund programme and Carlow Sports Partnership.

